# The Impact of Nutritional Status and Longitudinal Recovery of Motor and Cognitive Milestones in Internationally Adopted Children

**DOI:** 10.3390/ijerph8010105

**Published:** 2011-01-10

**Authors:** Hyun Park, Denise Bothe, Eva Holsinger, H. Lester Kirchner, Karen Olness, Anna Mandalakas

**Affiliations:** 1 Division of Developmental and Behavioral Pediatrics, Department of Pediatrics, University of California Irvine, 505 S. Main St., Suite 525, Orange, CA 92868, USA; E-Mail: hyunp@uci.edu; 2 For OC Kids Neurodevelopmental Center, 1915 W. Orangewood Ave, Suite 200, Orange, CA 92868, USA; 3 Department of Pediatrics, School of Medicine, Case Western Reserve University, 2109 Adelbert Rd., Cleveland, OH 44106, USA; 4 Division of Developmental Behavioral Pediatrics and Psychology, Rainbow Babies and Children’s Hospital, 10524 Euclid Avenue, Cleveland, OH 44106, USA; 5 Department of Pediatrics, School of Medicine, Case Western Reserve University, Cleveland, OH 44106, USA; E-Mails: soccernorsk@gmail.com (E.H.); kno@case.edu (K.O.); anna.mandalakas@case.edu (A.M.); 6 Henry Hood Centre for Health Research, Geisinger Health System, 100 N. Academy Ave., Danville, PA 17822, USA; E-Mail: hlkirchner@geisinger.edu

**Keywords:** malnutrition, international adoption, cognitive impairment, developmental delay, nutrition

## Abstract

Internationally adopted children often arrive from institutional settings where they have experienced medical, nutritional and psychosocial deprivation. This study uses a validated research assessment tool to prospectively assess the impact of baseline (immediately post adoption) nutritional status on fifty-eight children as measured by weight-for-age, height-for-age, weight-for-height and head circumference-for-age *z* scores, as a determinant of cognitive (MDI) and psychomotor development (PDI) scores longitudinally. A statistical model was developed to allow for different ages at time of initial assessment as well as variable intervals between follow up visits. The study results show that both acute and chronic measures of malnutrition significantly affect baseline developmental status as well as the rate of improvement in both MDI and PDI scores. This study contributes to the body of literature with its prospective nature, unique statistical model for longitudinal evaluation, and use of a validated assessment tool to assess outcomes.

## 1. Introduction

Children immigrating via international adoption have frequently lived in large institutions where their physical and emotional needs are not consistently met. Many of these children arrive from orphanages in China, Eastern Europe and the former Soviet Union which often have high child to caretaker ratios, limited access to health care, poorly maintained buildings, low paid staff and frequent relocation of children. Hence, it is common for children to experience psychosocial deprivation, poor nutrition, and infections which are well recognized risk factors for growth and cognitive deficits. While internationally adopted children (IAC) have several risk factors for poor growth and development, under-nutrition, including macro and micronutrient deficiencies, is a large factor. Physical growth and development of IAC has been a big topic of research in recent years. Studies have confirmed that children who have spent early time in institutionalized settings have signs of growth deficiency and developmental delays [1–5]. Some studies also compare growth and child development in foster care vs. orphanage settings. These results support the idea that being raised in a family-like setting such as foster care is better for nutrition, growth, attachment, and development [6,7].

The literature has also consistently described resiliency in IAC with respect to physical growth. Studies show that children who lived in institutions early in infancy and childhood display significant physical catch-up growth after adoption [7–10]. Comparison of studies illustrates that children who remain in deprived situations continue to be stunted, whereas children adopted into family settings show significant physical catch-up growth [8]. A large study of exclusively Romanian adoptees found that despite catch-up in height and weight, head circumference lagged behind several years after adoption [11]. Similarly, a meta-analysis including children from a variety of countries illustrated that IAC experience greater catch-up growth in height and weight as compared to modest catch-up in head circumference [10].

Developmental delays in IAC have also been documented [4]. Meta-analysis of this data demonstrates that developmental delays are positively associated with the length of time spent in an institution and age at adoption [11]. A few longitudinal studies of IAC from institutional settings describe dramatic catch up in developmental milestones beginning soon after adoption [3,9,11]. Many studies have been reviewed and consistently show an association between length of institutionalization and developmental catch up [12,13].

Very few studies describe the long term cognitive development of IAC. Studies of Korean [14] and Romanian children [11,15,16] showed that children eventually “catch up” in terms of having a normal range IQ, but may have deficits in specific areas, such as specific learning disabilities and executive function. Neuropsychological assessment has specifically identified cognitive deficiencies on tests of visual memory and attention, as well as visually mediated learning and inhibitory control in IAC [15].

Only 2 studies have used validated developmental assessment tools to describe IAC soon after adoption. Jacobs *et al.* used the Mullen Scales of Early Learning and Preschool Language Scale (PLS-4) to assess the development of IAC at time of arrival and again at 4–5 years of age; this study found significant delays at the time of adoption and considerable developmental catch-up in motor and language skills [17]. One cross-sectional study rigorously measured the association between nutritional status and development of IAC at the time of adoption [5] and found that lower z scores for height, weight and head circumference were associated with higher incidence of developmental delays in motor and language skills. Hence, we have used validated measures to longitudinally assess the association between nutritional status and motor and cognitive development in IAC.

## 2. Experimental Section

### 2.1. Procedures

From April 2001 to February 2002, we recruited children younger than 42 months of age who presented to the Adoption Health Service at Rainbow Babies and Children’s Hospital in Cleveland, Ohio for post-adoptive care within 2 months of immigration. This clinic provides comprehensive evaluation of internationally adopted children, including a complete history and physical examination, anthropometric measurements, developmental evaluation and laboratory testing as recommended for internationally adopted children [18]. Patients are routinely followed for post adoption care at 3, 6 and 12 months after their initial assessment.

After completion of informed consent and assessment of anthropometric measures during the medical visit, families were brought to a different room for Bayley Scales of Infant Development 2nd Edition (BSIDII) testing to improve validity of testing [19]. The BSIDII testing lasted 40–60 min and was administered prior to performing any laboratory work. At follow up clinical visits, the BSIDII was administered either before or after the medical visit and prior to obtaining any laboratory work. Families who did not return for follow up care were contacted by phone to arrange for follow up assessments.

This study was approved by the Institutional Review Board of University Hospitals of Cleveland.

### 2.2. Nutritional Assessment

With the use of standardized, calibrated measuring equipment, anthropometric measurements were obtained at each visit. Weight and height were obtained by trained nurses and head circumference by pediatricians. Recumbent length was measured in children who were under 18 months of age, while standing heights were measured in children older than 18 months. Length and height were rounded off to the nearest millimeter. Weight was rounded off to the nearest 100 g. Acute malnutrition (wasting) and chronic malnutrition (stunting) were assessed with moderate to severe acute malnutrition defined as a weight-for-height *z* score (WHZ) of less than or equal to −2 and moderate to severe chronic malnutrition defined as a height-for-age *z* score (HAZ) of less than or equal to −2. Weight-for-age *z* scores (WAZ) (a composite measure of acute and chronic malnutrition) and head circumference for age *z*-scores (HCZ) were also calculated and defined in a similar manner [20]. *Z* scores were calculated using the 2006 World Health Organization Child Growth Standards for children up to 5 years of age and the 2000 Centers for Disease Control and Prevention growth charts for children who were older than 5 years [21,22]. The expression in *z* scores uses SD of the reference distribution as the unit for a given height, length, or weight at specific ages. By convention, children with *z* scores between −3 and less than or equal to −2 are considered to have moderate malnutrition, and children with *z* scores of less than −3 are considered to have severe malnutrition [23].

For children born less than 38 weeks of gestation, an age adjustment (see statistical section) to the anthropometric measurements and the BSID scores was performed. Anemia was defined as venous blood hemoglobin level (hgb) less than 10.5 mg/dL which is two standard deviations below the mean value for infants and toddlers 6 months to 2 years of age.

### 2.3. Developmental Assessment

The developmental status of study subjects was measured at baseline and follow up visits using the BSID II. The BSID II is a widely used standardized developmental assessment and research tool used to document the differences between normal-risk children and those born at risk of developmental delay. It consists of three scales: the Mental Scale (MDI), Motor Scale (PDI), and Behavioral Rating Scale (BRS). The Mental Scales include items that assess memory, habituation, problem solving, early number concepts, generalization, classification, vocalizations, language and social skills. The Motor Scales assess control of key fine and gross motor groups. The BRS assesses the child’s behavior during the testing situation, which facilitates interpretation of the Mental and Motor Scales. Raw scores are converted to index scores. On the MDI and PDI, the mean score is 100 with a range of 50 to 150, and one standard deviation equals 15. In this study, children whose raw scores resulted in index scores lower than 50 were given a score of 49. Scores of children older than 42 months of age at the second visit were not considered in the statistical model, since there is no way to calculate an index score. All BSIDII testing was performed by one research assistant and one study investigator, who had previous experience administering the test to young children.

### 2.4. Statistics

To calculate the rate of change in MDI and PDI scores in a population without uniform age of enrollment and follow up time, it was necessary to use a statistical model to accommodate for these variations in the model. The linear mixed-effect model with a random subject effect was used to estimate the associations of age and anthropometric measures. Due to the variability in age at baseline each covariate was separated into cross-sectional and longitudinal effects. The cross-sectional effect describes the relationship of the outcome (e.g., MDI) to covariates at baseline, and the longitudinal effects describe the relationship of the change in outcome to change in the covariates. This is interpreted as the rate of catch-up in development.

For children born less than 38 weeks gestation an adjustment to their chronological age was performed. The correction subtracted the difference between 40 weeks and their gestational age from their chronological age. This was done for the baseline assessment to calculate the appropriate anthropometric z scores. Results are expressed as slopes from the regression model (*i.e.*, rate of change).

## 3. Results

A total of 58 internationally adopted children participated in the study. Thirty-four (58%) were females. Mean age was 17.6 months (range 4.7–39.5) at the time of enrollment. Children came from 11 different countries, mainly Russia (62%), followed by China (9%) and Ukraine (5%). Children came to the clinic on average after 19 days (range 2–69) of arrival in the USA. Twenty-two children were reported to be born prior to 38 weeks of gestation, while eight children did not have information regarding gestational age and were therefore assumed to have not been born prematurely. Four families had adopted 2 children at the same time; among those, two children were biological siblings. Thirty-three children (57%) returned for follow up testing. The median follow up time was 8 months (range 4.6–15.6). Families who declined to repeat the testing (N = 23) expressed reasons such as “both parents are working—too busy” and “my child is doing well”. Two families moved to a different state and were out of reach.

The majority of the children were undernourished at baseline as evidenced by 77%, 79%, 64%, and 90% of values less than zero for HCZ, HAZ, WHZ and WAZ, respectively. Specifically, z scores were less than −2.0 in 16%, 17%, 10 %, and 28% of children, respectively. There was no significant correlation between age of participants at baseline and HCZ, HAZ, WHZ or WAZ scores. There were 6 children (11.5%) with anemia (Hgb < 10.5).

Mean and median MDI and PDI scores for our study population were well below the mean of 100 for standardized populations (See Table 1). There was no significant difference in HCZ, HAZ, WHZ, WAZ and MDI between children who completed follow-up and children who only completed baseline assessment. Of note, children with only baseline assessments had a higher PDI (p = 0.04).

**Figure 1:**
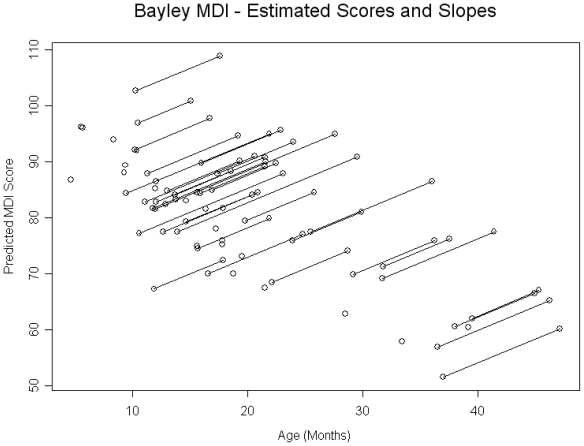
Bayley MDI-estimated scores and slopes.

With regard to baseline cognitive development, 29% of children had MDI scores < 70, and 59% had scores < 85. In a baseline model it was observed that cognitive development was inversely related to age of child at baseline, however the rate of catch-up was positively associated. For each month increase in age that the child was seen in the adoption clinic at baseline, the MDI was predicted to be 1.02 points lower (SE = 0.20, p < 0.0001). The MDI was then found to be 0.89 points higher for each month of age since the baseline visit (SE = 0.33, p = 0.0139) (See Figure 1). WAZ was positively and significantly associated with MDI while controlling for age of child. For each standard deviation decrease in WAZ, on average, the MDI score is expected to decrease by 2.95 points (SE = 1.32, p = 0.0327). A similar relationship was found for WHZ. For each standard deviation decrease in WHZ the average MDI score was decreased by 2.71 points (SE = 1.32, p = 0.0497). Gender, HAZ, HCZ, prematurity, and anemia were not significantly associated with MDI.

**Figure 2:**
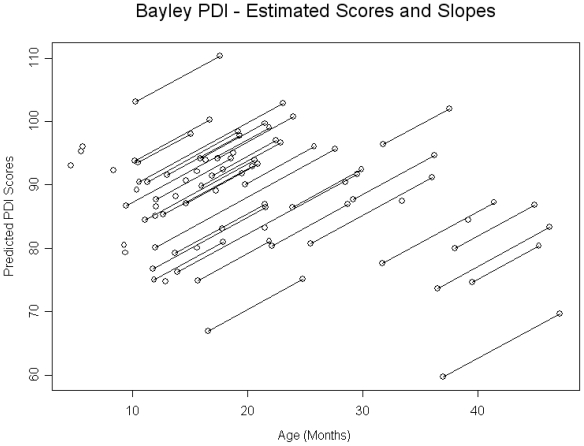
Bayley PDI-estimated scores and slopes.

With regard to gross and fine motor development, 22% of children had PDI scores < 70, and 48% had scores < 85. Age was found to be negatively related to PDI scores at baseline; however, the effect was not significant. For each month increase in age of child at baseline the PDI score was 0.32 points lower (SE = 0.24, p = 0.1991). The rate of catch up was significant such that for each month since the baseline visit the PDI score was 1.0 points greater (SE = 0.38, p = 0.0134) (See Figure 2).

After controlling for age, PDI score was significantly associated with baseline WAZ (p = 0.0003) and WHZ. Thus, for each SD decrease in WAZ, WHZ, and HCZ (*i.e.*, −1 to −2), the average PDI score decreased by 5.9, 4.9, and 3.7 points, respectively. No significant association was found between PDI scores and gender, HAZ, anemia or prematurity.

## 4. Discussion

Our study findings suggest that nutritional status has a significant impact on both cognitive and psychomotor development in IAC. This association was present at baseline and persisted longitudinally during periods of developmental catch-up. Using classical anthropometric measures, we demonstrated that the degree of children’s nutritional deprivation was directly correlated with developmental delays as measured by the BSIDII. These longitudinal associations suggest that malnutrition exerts a persistently negative effect on long term cognitive ability despite improvements in nutrition.

The results of our study indicate that children who are adopted from institutional environments have high rates of both malnutrition and developmental delay in the immediate post adoptive period. Specifically, WAZ, HAZ and HCZ were all associated with the baseline MDI and PDI scores of the BSIDII. Children in the study had MDI and PDI scores well below the mean of the general population, although the group as a whole did not fall into the range for developmental delay. These cross sectional results are consistent with previous studies of IAC that used less rigorous measures of psychomotor and cognitive development in IAC [4,11–13]. MDI and PDI scores both improved with time, reaching well into the mean range for the general population, with rate of improvement significantly related to markers of both acute and chronic malnutrition.

Our study offers new insight and enhances the existing literature. We prospectively evaluate developmental catch-up during the first year post adoption whereas previous studies have mainly been retrospective. In addition, we utilized a developmental assessment tool validated for research in infants and young children. Previous studies have used mainly developmental screening tools in the early post adoptive period. We have also created a statistical model that accommodates variations in age at recruitment and follow-up interval to enable estimation of the association between malnutrition and the rate of developmental catch-up longitudinally during the first year post adoption.

Although both cognitive and motor development delays were present in a large number of the children, it is notable that children had greater delays in cognition as measured by the BSIDII. Frequently initial evaluations of children may focus more on gross and fine motor development, but the importance of identifying and addressing possible cognitive delays from the onset must be emphasized. Children coming from an institutional setting have already lost countless opportunities to stimulate cognitive development prior to adoption, and it must be assumed that they have deficits to some degree until a thorough evaluation shows otherwise. Concurrent aggressive nutritional rehabilitation ought to accompany attention to cognitive and psychomotor development commencing immediately after adoption.

Our study clearly illustrates the persistent adverse effects of malnutrition on both the cognitive and psychomotor development of young children despite improvements in nutrition and physical growth during the first year of nutritional rehabilitation. Our findings are similar to the seminal work of Galler that showed an increased risk of learning disabilities and attention issues in children who experienced one episode of malnutrition during their first year of life [24–27]. We postulate that serial episodes of malnutrition extending beyond the first year of life, as found in our study cohort, are likely to be associated with a significant risk of long term cognitive impairment leading to subsequent learning disabilities that become evident during the school years. Recurrent nutritional deprivation early in life may lead to persistent and pervasive cognitive deficits that result in life-long challenges and prevent children from reaching their true potential. More research needs to be done to assess the best nutritional recommendations for children who have experienced under-nutrition early in life, including macro and micronutrients. For example, iron is one such nutrient that has been shown to be important in all areas of child development. Recent studies by Lozoff have shown a link between iron deficiency (with and without anemia) and developmental delays and cognitive impairments [28–31], and IAC have been found to have higher rates of iron deficiency with and without anemia [32]. The lack of significant association of anemia with MDI in our study may be in part related to children’s rapid ability to correct anemia, and by the time of presentation in the study anemia may have been resolved; in addition, children in our study were not assessed comprehensively for iron deficiency so those without anemia would not have been identified. Enhanced efforts to prevent malnutrition in young, developmentally vulnerable children are likely a wise investment as these efforts may mitigate the need for later costly intervention aimed at improving cognitive function.

We recognize the possible limitations in this study, including the inability to perform developmental assessments in a child’s native language and the widely varying countries of origin. Although completing the baseline assessment in English may have led to a slight overestimation of delays in cognitive development, studies using native language development in Romanian orphans have found increase in verbal IQ with improved nutritional status comparable to our study findings [7]. Although pragmatically challenging, inclusion of children from a variety of cultural and ethnic backgrounds improves the generalizability of our findings. Although we have adjusted chronologic age to account for reported prematurity, the gestational age reported in medical referral documents is often based on the child’s birth weight; hence, age values used in our study may have minor inaccuracies.

The low rate of follow-up for developmental assessments, particularly in children with greater motor skills, represents a clinical situation where adoptive parents perceive that their adoptees are “doing fine and do not need a follow up visit”. This parental behavior commonly introduces a bias into clinical studies that favors inclusion of children with more significant delay, which may be particularly true for delays related to gross and fine motor function.

While we studied primarily growth and nutrition related to development, there are many risk factors for growth and cognitive impairment. Children living within an institutional environment prior to adoption are deprived in a number of areas. Growth requires both an optimal nutritional as well as psychosocial and neuroendocrine environment. Endocrinology studies have demonstrated growth failure and recovery associated with reversible suppression of the growth hormone-IGF-1 axis (GH-IGF-1 axis) in children who are socially and not necessarily nutritionally deprived [33,34]. Also children under extreme stress characterized by high cortisol levels have been found to have signs of cell loss in certain brain areas, such as the hippocampus, indicating that chronic exposure to stress during institutionalization may be detrimental to cognitive function in children [35]. While we have shown that nutrition is associated with developmental outcomes in IA children, psychosocial deprivation and stress frequently experienced before adoption and improved care giving environments experienced after adoption both likely influence subsequent growth and cognitive development after adoption.

Finally, we recognized that our study only used baseline nutritional status to predict changes in developmental outcome. Future studies examining longitudinal changes in nutritional status and brain development and cognition over longer periods of time are needed to assess the long term effects of poor nutrition on cognitive functioning.

## 5. Conclusions

International adoptees are a vulnerable group of children who experience high rates of malnutrition. Our study demonstrates that children with signs of poor nutrition as measured by growth also have associated high rates of developmental delays. In addition, these children have early signs of cognitive deficits. Longitudinally, these children were able to show motor and cognitive gains toward the normal range with improvements in their nutritional status. While maximizing nutrition after periods of under-nutrition is crucial to support cognitive recovery, ensuring adequate and consistent nutrition for vulnerable children with developing minds to prevent cognitive injury is paramount.

## Figures and Tables

**Table 1:** Baseline and Follow-up Growth Parameters and Bayley scores.

	Baseline (N = 58)		Follow Up (N = 33)	
	Mean (SD)	Median (IQR)**	Mean (SD)	Median (IQR)**
Age (months)	17.6 (8.9)	15.6 (11.8, 9.8)	26.9 (9.5)	22.9 (20.6, 29.9)
Months between visits			8. 0 (2.8)	6.9 (6.2, 9.7)
MDI	78.4 (16.0)	80.5 (67.0, 90.0)	88.0 (18.5)	88.0 (76.0, 100.0)
PDI	84.9 (17.8)	85.5 (70.0, 99.0)	94.9 (16.5)	97.5 (87.0, 107.0)
WAZ*	−1.4 (1.3)	−1.3 (−2.1, −0.4)		
HAZ*	−1.1 (1.1)	−1.0 (−1.7, −0.2)		
WHZ*	−0.5 (1.3)	−0.5 (1.5, 0.6)		
HCZ*	−0.8 (1.2)	−0.8 (−1.6, 0.0)		

* Nutritional measurements from baseline visit;

** IQR = inter-quartile range.

## References

[1] Albers LH Johnson DE Hostetter MK Iverson S Miller LC Health of children adopted from the former Soviet Union and Eastern Europe. Comparison with preadoptive medical records JAMA 1997 278 922 924 9302245

[2] Johnson DE Miller LC Iverson S Thomas W Franchino B Dole K Kiernan MT Georgieff MK Hostetter MK The health of children adopted from Romania JAMA 1992 268 3446 3451 1281241

[3] Lien NM Meyer KK Winick M Early malnutrition and “late” adoption: A study of their effects on the development of Korean orphans adopted into American families Am. J. Clin. Nutr 1977 30 1734 1739 910749 10.1093/ajcn/30.10.1734

[4] MacLean K The impact of institutionalization on child development Dev. Psychopathol 2003 15 853 884 14984130 10.1017/s0954579403000415

[5] Miller LC Kiernan MT Mathers MI Klein-Gitelman M Developmental and nutritional status of internationally adopted children Arch. Pediatr. Adolesc. Med 1995 149 40 44 7827658 10.1001/archpedi.1995.02170130042009

[6] van den Dries L Juffer F van Ijzendoorn MH Bakermans-Kranenburg MJ Infants’ physical and cognitive development after international adoption from foster care or institutions in China J. Dev. Behav. Pediatr 2010 31 144 150 20110827 10.1097/DBP.0b013e3181cdaa3a

[7] Johnson DE Guthrie D Smyke AT Koga SF Fox NA Zeanah CH Nelson CA 3rd Growth and associations between auxology, caregiving environment, and cognition in socially deprived Romanian children randomized to foster vs ongoing institutional care Arch. Pediatr. Adolesc. Med 2010 164 507 516 20368481 10.1001/archpediatrics.2010.56 PMC4126580

[8] Martorell R Khan LK Schroeder DG Reversibility of stunting: Epidemiological findings in children from developing countries Eur. J. Clin. Nutr 1994 48 S45 S57 8005090

[9] Benoit TC Jocelyn LJ Moddemann DM Embree JE Romanian adoption. The Manitoba experience Arch. Pediatr. Adolesc. Med 1996 150 1278 1282 8954000 10.1001/archpedi.1996.02170370056008

[10] Van Ijzendoorn MH Bakermans-Kranenburg MJ Juffer F Plasticity of growth in height, weight, and head circumference: Meta-analytic evidence of massive catch-up after international adoption J. Dev. Behav. Pediatr 2007 28 334 343 17700087 10.1097/DBP.0b013e31811320aa

[11] Rutter M Developmental catch-up, and deficit, following adoption after severe global early privation. English and Romanian Adoptees (ERA) Study Team J. Child. Psychol. Psychiatry 1998 39 465 476 9599775

[12] Judge S Developmental recovery and deficit in children adopted from Eastern European orphanages Child. Psychiatry Hum. Dev 2003 34 49 62 14518623 10.1023/a:1025302025694

[13] Weitzman C Albers L Long-term developmental, behavioral, and attachment outcomes after international adoption Pediatr. Clin. North Am 2005 52 1395 1419 16154469 10.1016/j.pcl.2005.06.009

[14] Winick M Meyer KK Harris RC Malnutrition and environmental enrichment by early adoption Science 1975 190 1173 1175 1198103 10.1126/science.1198103

[15] Pollak SD Nelson CA Schlaak MF Roeber BJ Wewerka SS Wiik KL Frenn KA Loman MM Gunnar MR Neurodevelopmental effects of early deprivation in postinstitutionalized children Child. Dev 2010 81 224 236 20331664 10.1111/j.1467-8624.2009.01391.x PMC2846096

[16] Rutter M Sonuga-Barke EJ X. Conclusions: Overview of findings from the era study, inferences, and research implications Monogr. Soc. Res. Child. Dev 2010 75 212 229 20500640 10.1111/j.1540-5834.2010.00557.x

[17] Jacobs E Miller LC Tirella LG Developmental and behavioral performance of internationally adopted preschoolers: A pilot study Child. Psychiatry Hum. Dev 2010 41 15 29 19593639 10.1007/s10578-009-0149-6

[18] Red Book American Academy of Pediatrics Elk Grove Village, IL, USA 2009

[19] Bayley N Bayley Scales of Infant Development Manual2nd ed The Psychological Corporation San Antonio, TX, USA 1993

[20] Arbelot A Nutrition Guidelines1st ed Medecins Sans Frontieres Paris, France 1995

[21] CDC Growth Charts Centers for Disease Control and Prevention Atlanta, GA, USA 2000

[22] WHO child growth standards based on length, height, weight, and age Acta Pediatr 2006 450 76 85 10.1111/j.1651-2227.2006.tb02378.x 16817681

[23] WHO Working Group Use and interpretation of anthropometric indicators of nutritional status Bull. World Health Organ 1986 64 929 941 3493862 PMC2490974

[24] Galler JR Malnutrition—a neglected cause of learning failure Postgrad. Med 1986 80 225 228 3763526 10.1080/00325481.1986.11699571

[25] Galler JR Ramsey F A follow-up study of the influence of early malnutrition on development: V. Delayed development of conservation (Piaget) J. Am. Acad. Child Adolesc. Psychiatry 1987 26 23 27 3583996 10.1097/00004583-198701000-00005

[26] Galler JR Ramsey F Solimano G The influence of early malnutrition on subsequent behavioral development III. Learning disabilities as a sequel to malnutrition Pediatr. Res 1984 18 309 313 6718088 10.1203/00006450-198404000-00001

[27] Galler JR Ramsey F Solimano G Influence of early malnutrition on subsequent behavioral development. V. Child’s behavior at home J. Am. Acad. Child. Psychiatry 1985 24 58 64 3968347 10.1016/s0002-7138(09)60410-6

[28] Lukowski AF Koss M Burden MJ Jonides J Nelson CA Kaciroti N Jimenez E Lozoff B Iron deficiency in infancy and neurocognitive functioning at 19 years: Evidence of long-term deficits in executive function and recognition memory Nutr. Neurosci 2010 13 54 70 20406573 10.1179/147683010X12611460763689 PMC3151652

[29] Carter RC Jacobson JL Burden MJ Armony-Sivan R Dodge NC Angelilli ML Lozoff B Jacobson SW Iron deficiency anemia and cognitive function in infancy Pediatrics 2010 126 e427 e434 20660551 10.1542/peds.2009-2097 PMC3235644

[30] Lozoff B Clark KM Jing Y Armony-Sivan R Angelilli ML Jacobson SW Dose-response relationships between iron deficiency with or without anemia and infant social-emotional behavior J. Pediatr 2008 152 696 702 18410777 10.1016/j.jpeds.2007.09.048 PMC2391001

[31] Shafir T Angulo-Barroso R Jing Y Angelilli ML Jacobson SW Lozoff B Iron deficiency and infant motor development Early Hum. Dev 2008 84 479 485 18272298 10.1016/j.earlhumdev.2007.12.009 PMC3144491

[32] Fuglestad AJ Lehmann AE Kroupina MG Petryk A Miller BS Iverson SL Johnson DE Georgieff MK Iron deficiency in international adoptees from Eastern Europe J. Pediatr 2008 153 272 277 18534235 10.1016/j.jpeds.2008.02.048

[33] Saitoh H Kamoda T Fukushima T The status of the GH-IGF-I axis in a child with psychosocial short stature J. Pediatr. Endocrinol. Metab 2003 16 439 441 12705370 10.1515/jpem.2003.16.3.439

[34] Nieves-Rivera F Gonzalez de Pijem L Mirabal B Reversible growth failure among Hispanic children: instances of psychosocial short stature P. R. Health Sci. J 1998 17 107 112 9803487

[35] Lombroso PJ Sapolsky R Development of the cerebral cortex: XII. Stress and brain development: I J. Am. Acad. Child. Adolesc. Psychiatry 1998 37 1337 1339 9847508 10.1097/00004583-199812000-00019

